# The shallow cognitive map hypothesis: A hippocampal framework for thought disorder in schizophrenia

**DOI:** 10.1038/s41537-022-00247-7

**Published:** 2022-04-07

**Authors:** Ayesha Musa, Safia Khan, Minahil Mujahid, Mohamady El-Gaby

**Affiliations:** 1 Green Templeton College, University of Oxford, Oxford, OX2 6HG UK; 2 St Anne’s college, University of Oxford, Oxford, OX2 6HS UK; 3 Nuffield Department of Clinical Neurosciences, University of Oxford, Oxford, OX1 3SR UK

**Keywords:** Schizophrenia, Schizophrenia, Psychosis, Neural circuits, Psychosis

## Abstract

Memories are not formed in isolation. They are associated and organized into relational knowledge structures that allow coherent thought. Failure to express such coherent thought is a key hallmark of Schizophrenia. Here we explore the hypothesis that thought disorder arises from disorganized Hippocampal cognitive maps. In doing so, we combine insights from two key lines of investigation, one concerning the neural signatures of cognitive mapping, and another that seeks to understand lower-level cellular mechanisms of cognition within a dynamical systems framework. Specifically, we propose that multiple distinct pathological pathways converge on the shallowing of Hippocampal attractors, giving rise to disorganized Hippocampal cognitive maps and driving conceptual disorganization. We discuss the available evidence at the computational, behavioural, network, and cellular levels. We also outline testable predictions from this framework, including how it could unify major chemical and psychological theories of schizophrenia and how it can provide a rationale for understanding the aetiology and treatment of the disease.

## Introduction

*“I think someone’s infiltrated my copies of the cases. We’ve got to case the joint. I don’t believe in joints, but they do hold your body together”.* From a patient with Schizophrenia^1^.

The ability to translate our experiences into coherent thoughts is central to our daily function. A stark reminder of this comes from appreciating conceptual disorganization (also known as positive formal thought disorder), one of the key hallmarks of schizophrenia. Patients exhibit incoherent, undirected speech (tangentiality), often making inappropriate associations between unrelated or loosely related experiences and concepts (loosening of associations/derailment)^2,3^. This can have a debilitating effect on their ability to communicate and function in society. A key challenge in Schizophrenia research has been to understand the ontogeny of such disorganization, in the hope of developing more effective treatments. To achieve this, it is necessary to first understand how our experiences are associated to form organized models of the world, which in turn gives rise to organized thought.

One of the key breakthroughs in psychology has been the conceptualization of the cognitive map. This theory posits that animals not only form associations between directly experienced events, but also organize such relationships into coherent structures that enable flexible inference^4^. Animals can find shortcuts in mazes along paths they have never traversed, after mapping the relationships between the different locations they have visited^4^. Analogous inferences can be made in the non-spatial domain, where distinct experiences can be associated to infer new links that together build a coherent model of the world^5–7^. Knowing that A > B and B > C allows inferring that A > C, but only if memories of the A-B and B-C associations are *organized* on a line. In the search for a neural basis of such cognitive mapping, research on the Hippocampus and associated regions in the medial temporal lobe has been particularly fruitful. Lesion, inactivation, neuroimaging, and electrophysiological studies across multiple mammalian species implicate such circuits in forming relational maps of the world and enabling inferences^8,9^. That Hippocampal pathology is also strongly implicated in schizophrenia raises the possibility that thought disorder may be the result of disorganized Hippocampal cognitive maps. Here we review the latest evidence suggesting a convergence of multiple distinct mechanisms and aetiological pathways on disorganized Hippocampal maps. We begin by outlining and motivating the *shallow cognitive map* hypothesis. We then explore three key predictions of this framework and outline evidence from schizophrenia patients and animal models speaking to each. We note that schizophrenia is not a solely Hippocampal disorder, and pathologies in areas such as the Prefrontal cortex have been strongly implicated in the disease^10,11^. Our focus here is on the Hippocampus, since it is there that we understand the most about the mechanisms behind cognitive mapping. Nevertheless, insights from exploring shallow cognitive maps in the Hippocampus may well generalize to other cortical drivers of the disease. We have also focused on positive formal thought disorder, or conceptual disorganization, as opposed to negative formal thought disorder (poverty of speech and poverty of content)^12^.

### The shallow cognitive map hypothesis of thought disorder

**Fig. 1:**
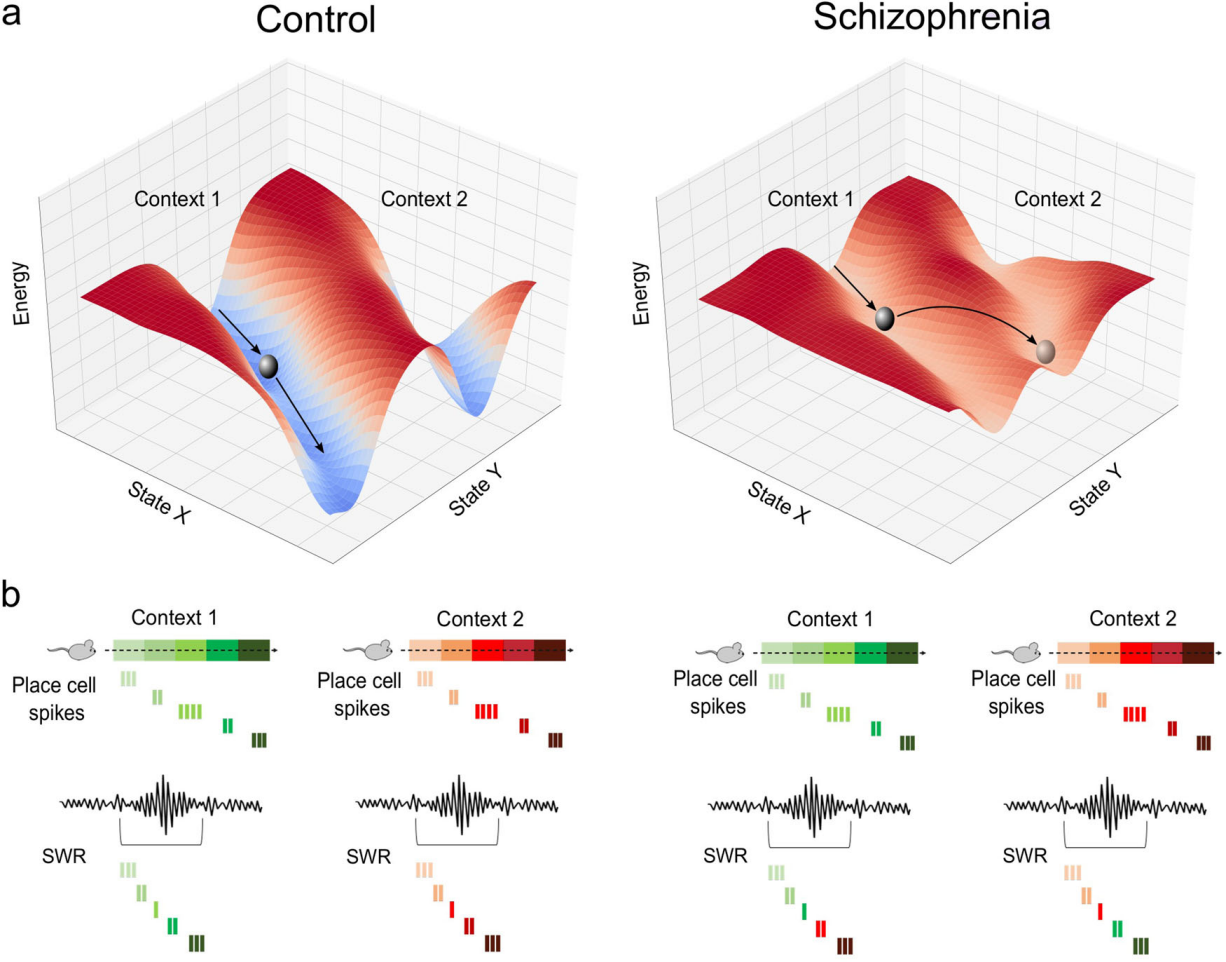
Shallow attractors and disorganized cognitive maps in Schizophrenia. **a** Hypothetical neural state space representing the stability of distinct ensemble activity patterns (only two state dimensions, *X* and *Y*, are shown for simplicity). In controls, attractors (valleys) representing distinct contexts are stable and well-separated by relatively low stability states (hills). This stability is hypothesized to be reduced in Schizophrenia, whereby attractors for distinct contexts are relatively shallower. **b** A hypothetical instantiation of shallow attractors in Hippocampal cognitive mapping. In controls, place cell activity during learning is replayed during Sharp Wave Ripples (SWRs) that occur at rest. Sequences of place cell activity from different contexts are replayed separately. This reflects deep attractors, requiring greater jumps in energy barriers to move between attractors of distinct memories. In Schizophrenia, place cell activity during learning is replayed during SWRs, however, replay is disordered. This may arise due to shallow attractors, in which energy barriers are smaller and easier to cross, making it easier to switch between memories of unrelated contexts during retrieval and hence giving rise to disordered thought.

How does positive formal thought disorder arise in schizophrenia? One possible answer comes from considering the dynamical systems framework, especially the concept of attractors. Attractors are stable states of activity towards which a complex system (e.g. a neural network) evolves. Several stable states, representing distinct memories, may exist within the same network. A particular sensory cue biases the network to evolve towards a particular attractor state, one that was perhaps associated with the cue during learning. Owing to its stability, the network persists within an attractor state beyond the initial stimulus, thereby facilitating faithful memory retrieval^13^. Such attractors can be discrete, reflecting the activity of a defined assembly of neurons^13^. They can also be continuous, wherein the network moves between a constrained set of states (or assemblies) with minimal perturbation^14,15^. Attractor dynamics can be implemented by recurrent excitatory (glutamatergic) synaptic connectivity, which promotes positive feedback and hence persistent activity amongst assemblies of strongly connected principal neurons. GABAergic interneurons control such activity, in part by inhibiting the spread of activity *between* assemblies^16^. This is essential in defining the attractor “valley” (Fig. 1a, left), reducing the prevalence of “jumps” between distinct attractors. Learning can stabilize existing states or generate new stable states through promoting synaptic (and cellular) plasticity in the network. The greater the *relative* depth (stability) of the attractor state, the longer a network can persist there, and hence the lower the likelihood of jumping between states due to distractors or intrinsic noise. This is proposed to ensure the sequential retrieval of memories, and hence the flow of thoughts, is both coherent and goal appropriate. It is hypothesised that shallow cortical attractors could give rise to thought disorder (especially tangentiality and derailment) by reducing the threshold for switching between attractor states^17–19^. We extend this idea with a specific emphasis on Hippocampal attractors involved in cognitive mapping.

Hippocampal cognitive mapping is particularly implicated in forming internal models that represent the links between memories, even when all the links have not been directly experienced^7,8^. Such “relational” mapping of disparate elements of experience is central to internal cognitive capacities such as imagery, inference, and thought^20^. However, dysregulated, excessive, or inappropriate associations may give rise to disorganized and debilitating thought. As the quote at the start of this article illustrates, such disorganization need not affect directly experienced associations. A patient can accurately retrieve the association between “joint” and “body” while making indirect associations between loosely related conjunctions. We propose that the shallowing of attractors in the Hippocampus may have the specific effect of disrupting the formation and/or retrieval of Hippocampus-dependent cognitive maps, and hence give rise to the aberrant associations expressed in thought disorder. During memory formation, this lowered threshold for jumping between attractors could alter the integrity of the relational memory stored, by permitting aberrant Hebbian associations between unrelated memories. During memory retrieval, the reduced stability of attractors means the threshold for switching between attractor-defined memories is lowered (Fig. 1a). In both cases, the end result would be the generation of disorganized maps that inappropriately relate separate events or memories, with this disrupted relational mapping driving thought disorder. We, therefore, propose that:

***Positive Formal Thought disorder in schizophrenia is mediated by disorganized cognitive maps resulting from the shallowing of neural attractors in the Hippocampus***

By considering the phenomenology and mechanisms underlying cognitive mapping in light of this hypothesis, a number of predictions arise. Below we outline these predictions, critically assess existing evidence pertaining to each and, where appropriate, suggest future experiments that address the current gaps.

### Prediction 1 – Positive Formal Thought disorder is associated with disorganized Hippocampal relational representations

A key hallmark of cognitive mapping in the Hippocampus is the phenomena of replay. This classically refers to sequential reactivation of spatial representations during offline rest/sleep that is congruent with the spatial structure of the environment, with adjacent locations being reactivated contiguously in the absence of the relevant sensory input^21^. In the Hippocampus, this replay typically occurs during brief periods of high neural synchrony characterized by sharp-wave ripples (SWRs; irregular deflections (sharp waves) associated with 140–200 Hz oscillations (ripples)) in the local field potential generated during slow-wave sleep or periods of awake immobility^22^. That this replay occurs in the absence of structured experience suggests an *internal* organization of representations, reminiscent of Tolman’s postulated cognitive maps^4^. Moreover, such SWR-associated replay has been implicated in memory retrieval in humans^23,24^ and rodents^25^ (but see Gillespie et al. 2021^26^). Similarly, theta oscillations occurring during online locomotion in rodents are associated with the ordered firing of place cells that represent the spatial organization of visited locations, albeit in a temporally compressed manner^27^. Such “theta sequences” are also implicated in the encoding and retrieval of memories^27^. The sequential reactivation of representations in both replay and theta sequences can be (re)framed in terms of attractor dynamics^28^. A replay event can be understood as a trajectory through an attractor valley that encodes the relational structure of a particular state-space in the outside world. States of the world that are more closely related are encoded closer on the neural activity space, and hence reactivated more closely in time, than more distantly related states. Crucially, such replay is not merely a recapitulation of experienced associations. States that have not been explicitly experienced together, but whose close relationship can be inferred by understanding the relational structure of the state space, are contiguously replayed^29,30^. This phenomenon is proposed to underlie the process of *relational* inference itself, allowing coherent thoughts and insights to emerge from explicit memories in the healthy mind^31^. This could be achieved by selectively lowering the threshold for transitions between attractor states representing distinct but related memories, effectively shallowing the attractor landscape only for transitions between these memories. If this process is dysregulated it would manifest as disorganized replay, where parts of sequences representing unrelated (or loosely related) memories are excessively and inappropriately intermixed (Fig. 1b). This may be especially associated with the positive (psychotic) state and particularly with conceptual disorganization.

Emerging evidence from patients and animal models suggests that offline replay is impaired in schizophrenia. In a recent study, Nour et al used Magnetoencephalography (MEG) to investigate offline replay of task representations in schizophrenia patients learning a structural inference task^32^. The authors used a task that involved learning a sequence of visual cues from separately experienced 1-step transitions (e.g. A->B; B->C…etc). While patients were unimpaired in remembering such 1-step transitions, they were impaired in using such experiences to transitively infer ordinal relationships that had not been explicitly observed (e.g. A->C)^32^. This could not be explained by more basic deficits in working memory or rule comprehension. Such an impairment is consistent with earlier results showing selective impairments in relational processing but not retrieval of directly experienced associations in schizophrenia^33^. These studies suggest that schizophrenia-associated impairments in relational knowledge can be dissociated from lower level deficits in associative memory. Crucially, Nour et al show that such a behavioural impairment was concomitant with impaired spontaneous (off-task) replay of sequential relationships between items. In both patients and controls, this replay was coincident with increased Hippocampal ripple power. However, schizophrenia patients exhibited an augmented ripple power relative to controls. This combination of impaired replay and enhanced Hippocampal ripple power mirrors that previously observed in mouse models of schizophrenia^34–36^, and is consistent with a strong involvement of Hippocampal offline replay in the expression of schizophrenia symptoms.

A number of key questions remain. First, whether *Hippocampal* replay is selectively impaired in human patients is unclear from the MEG analysis. Concordance with Hippocampal ripples suggests a Hippocampal involvement. This may reflect impaired replay in the Hippocampus, as seen in mouse models^34,35^. Alternatively, the observed human deficits could reflect impaired cortical replay, which can co-occur with Hippocampal ripples^37^. Second, while results from humans and animal models show *impaired* replay associated with schizophrenia, they fall short of showing *disorganized* replay. Replay impairment may simply reflect a loss of function, in which relational associations are weak or absent. Alternatively, it may represent a disorganization, where aberrant relational associations are made resulting in parts of distinct sequences being erroneously stitched together, as we propose here (Fig. 1b). Nour et al. did not find evidence for any specific instance of such disorganized replay. Such an absence of evidence may reflect methodological constraints of testing for the presence of a particular pattern of disorganized replay^38^. In addition, the replay was assessed in an off-task rest session. It is possible that replay (or other forms of organized, sequential activity, such as theta sequences) is disorganized during the process of inference itself in schizophrenic patients. Consistent with this, the authors find evidence for disorganized representations *during* task performance in schizophrenia^32^. Using representational similarity analysis in the visually evoked MEG data from a post-learning task, the authors report that controls exhibited representations that were more similar for items in equivalent positions across two sequences. In contrast, schizophrenia patients exhibited representational similarity patterns that systematically confused adjacent items both within and across sequences. Such a deficit is consistent with our proposal of disorganized cognitive mapping in schizophrenia. Third, it is not clear whether and how the impaired replay and representations are related to thought disorder. Nour et al find that both the strength of abstract position representations and replay are positively correlated with sequence learning in patients and controls. This may hint at a relationship to ordered thought. However, the authors did not find a relationship between the severity of positive symptoms, as assessed using the Positive and Negative Symptom Scale (PANSS), and behavioural/neural signatures of sequence learning. This may reflect the typically narrow and low range of symptom severity of patients that can be recruited to such studies. In particular, patients with severe thought disorder are typically excluded from such studies due to ethical considerations. It may also reflect the assessment of replay during off-task periods. On-task replay may be more closely linked to symptoms, especially thought disorder. A related question is the extent to which online theta sequences are impaired in schizophrenia. A plethora of studies point to the reduction in the power of theta and associated gamma (25–100 Hz) oscillations in schizophrenia^39^, rhythms strongly implicated in temporally organizing neural ensembles and cognitive mapping functions^40^. Moreover, findings from animal models of schizophrenia are consistent with aberrant theta-based organization of neural firing^41^. Whether and how these relate to psychotic symptoms in patients remains unclear.

The study by Nour et al provides unique insight into the role of cognitive mapping in schizophrenia. Future experiments will need to clarify whether it is the Hippocampal sequences that are impaired, or whether Hippocampal involvement relates to its permissive role in the generation of cortical sequences, e.g. via SWR-mediated Hippocampal–cortical interactions^42^. Combining the spatial resolution of functional magnetic resonance imaging (fMRI) with temporal resolution of MEG^43^ could allow investigating the relative roles of Hippocampal and cortical replay deficits in schizophrenia. This focus on replay and representations may explain discrepancies in literature describing the relationship between gross Hippocampal activity and psychotic symptoms. Studies using positron emission tomography and cerebral blood volume measurements with MRI show increased resting-state Hippocampal activity in schizophrenia patients^44–46^. This hyperactivity is positively correlated with psychotic symptoms^46^ and could predict progression from prodromal to psychotic states^45^. Conversely, studies in patients performing Hippocampus dependent tasks suggest reduced Hippocampal activity. In a task where patients were required to navigate towards goals in a virtual town, both contextual binding performance and fMRI-deduced Hippocampal/paraHippocampal activity were significantly decreased compared to controls^47^. While several factors may explain such a discrepancy (e.g., cross-study differences in medication status and disease classification/stage), an intriguing possibility arises from considering differences in behavioural state. Increases in offline Hippocampal activity and ripple power could reflect a compensatory effect that counteracts an online loss of function. Indeed, Nour et al find that, while Hippocampal ripple power is enhanced in schizophrenia patients, ripple power during post-learning rest is actually positively correlated with subsequently measured neural signatures of sequence learning in patients (but not controls^32^). This is consistent with enhanced offline ripple activity compensating for the impaired relational processing observed in this task, perhaps by promoting cortical replay. If such an enhancement of activity is compensatory then one would predict it would differ depending on disease state and medication, further explaining discrepancies in the literature. These considerations highlight the need to move beyond simple descriptions of changes in overall Hippocampal activity and towards an understanding of the representational and dynamical aspects of Hippocampal activity in relation to defined symptoms. They also implore the inclusion and careful stratification of symptomatically broad cohorts of patients in future studies, in order to avoid confounds relating to symptom heterogeneity. In particular, it will be important to investigate the link between disorganized Hippocampal cognitive maps and conceptual disorganization, especially derailment and tangentiality. Our framework predicts a positive correlation between conceptual disorganization score on the PANSS and (i) behavioural impairment in relational memory tasks (ii) neural representations that systematically confuse adjacent items across sequences (iii) the degree to which *awake* replay shows jumps between distinct sequences representing distinct relational sets (e.g. distinct spatial contexts). Overall, the hippocampal replay deficits reported in schizophrenia patients and models implicate aberrant *internal* Hippocampal relational mapping in schizophrenia. However, a more explicit assessment of the nature (disorganization versus loss of function) and timing (rest versus task engagement) of replay impairments and its relationship to the expression of thought disorder is needed to thoroughly assess our predictions.

### Prediction 2 - Hippocampal circuit pathology in schizophrenia converges on the shallowing of Hippocampal cognitive maps

A range of Hippocampal pathologies has been implicated in schizophrenia. These include reduced Hippocampal inhibition^48^, both decreased^49,50^ and increased^51^ long-term potentiation (LTP) at CA3-CA1 synapses, impaired pattern separation in the dentate gyrus/CA3^52,53^, enhanced pattern completion in the CA3^52,54^, CA3-associated aberrant salience^55^ and impaired goal representations in the CA1^36^. Here we highlight how these seemingly disparate circuit-level pathologies could all converge on the shallowing of Hippocampal attractors and disorganization of cognitive maps. In addition, we provide pointers for how such disorganized cognitive mapping in the Hippocampus could relate to schizophrenia-related pathologies in cortical regions.

### Imbalance of pattern separation and pattern completion

**Fig. 2:**
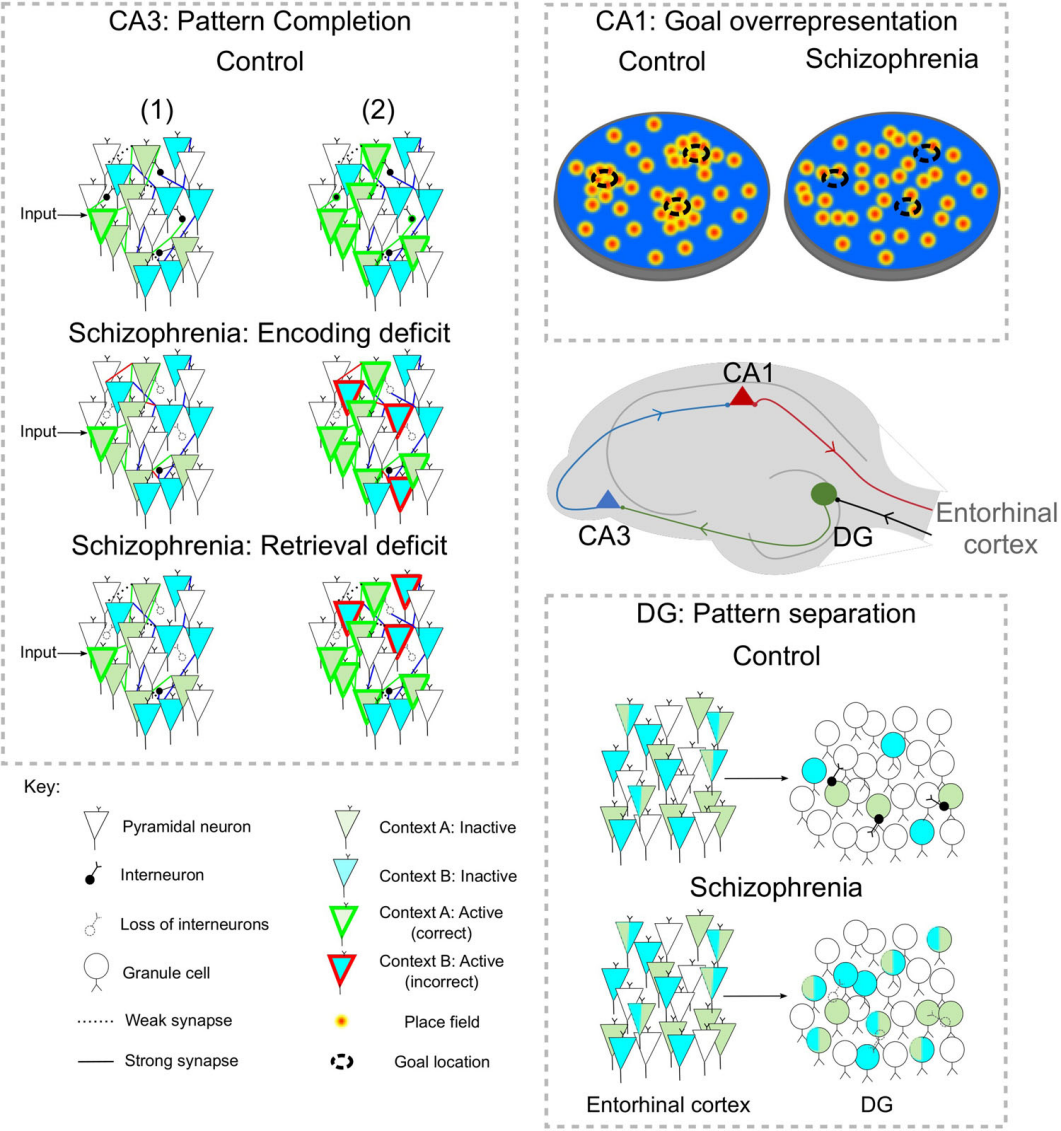
Hippocampal circuit elements and computations implicated in Schizophrenia. *Hippocampal subfields* and classical tri-synaptic connectivity (middle right): Impairments at each subfield could contribute to disorganized cognitive mapping. *Pattern separation* in the dentate gyrus (DG; bottom right panel): Experience of two different contexts results in activation of distinct but overlapping neuron groups in the entorhinal cortex. Sparse firing of dentate gyrus neurons reduces overlap in the representation of context A and B, allowing pattern separation in the dentate gyrus. In Schizophrenia, pattern separation in the dentate gyrus is reduced, reflected by increased overlap in representations of each context in the dentate gyrus. This is in part due to loss of inhibitory interneurons. *Pattern completion* in the CA3 (left panel): In controls, highly selective recurrent connectivity ensures that partial cues (1: top) activate context-selective neurons (2: top). Excessive pattern completion in Schizophrenia may arise through an encoding problem. Here, excessive connections are formed resulting in strong synaptic connections between neurons encoding different contexts (red lines connecting context A and context B neurons), which may arise due to dysregulated LTP (1: middle). When partial cues from one context are presented (1: middle) the activation of neurons encoding context A (green outline), would also result in the erroneous activation of neurons encoding context B (2: middle; red outline). This activation of neurons representing an irrelevant context could underlie thought disorder. Alternatively, a retrieval problem would mean that, when partial cues activate a neuron encoding context A (1: bottom: green outline), a loss of reciprocal inhibition due to interneuron loss, would result in erroneous activation of neurons encoding context B (2: bottom; red outline) in patients. This is a retrieval deficit as it can occur even in the absence of selective potentiation of synaptic connections between context A and context B neurons. *Overrepresentation of goal locations* by place cells in area CA1 (top right): Place cells accumulate around goal locations in controls. This overrepresentation is lost when NMDA receptors are blocked and in genetic models of Schizophrenia. This could be understood as a loss of attractors towards goal locations, resulting in disorganized goal-directed behaviour and thought.

The integrity of cognitive maps necessitates forming appropriate associations between related memories. This in turn relies on the balance between two processes: pattern separation (PS) and pattern completion (PC). An imbalance in PS and PC towards reduced PS and enhanced PC is hypothesized to underlie disorganized memories in schizophrenia (Fig. 2)^52^.

In PS, similar but unrelated memories are separated in order to avoid interference. Neurons in the dentate gyrus, a key Hippocampal input region, are implicated in this process along with downstream CA3 neurons^56,57^. Conversely, PC allows linking memory items in order to retrieve a whole memory from partial cues. An analogous process allows linking distinct but related memories in order to infer novel relationships^6^. Strong recurrent excitatory connections between CA3 neurons are implicated in this process^58,59^. Das et al report significant impairments in pattern separation performance in schizophrenic patients, without a significant basic difference in recognition memory compared to matched controls^60^. However, a later study by Martinelli & Shergill reported an impairment in familiarisation and visual discrimination ^61^ in the same tasks employed by Das et al., suggesting that the observed impairments could be explained by lower-level deficits, rather than a selective deficit in pattern separation. A meta-analysis of effects on recognition memory across multiple studies suggests that chronicity (i.e., duration of illness) and (to a lesser extent) PANSS score are key factors: more chronic or pronounced disease is associated with stronger recognition memory deficits^62^. Indeed, patients in the Das et al. and Martinelli & Shergill scored on average 38.82 and 83.92 on the PANSS respectively. This raises the possibility that early (and pre-clinical) stages of schizophrenia could present with more selective deficits in cognitive mapping while the disease may progress to produce low-level deficits in recognition memory. The reason for this progression is currently unclear but it may arise from increased severity of the pathological changes leading to progressive shallowing of attractors across disparate circuits. This coupled with the higher stability of attractors representing memories for experienced associations compared to those for inferred links could explain the progression from selective relational impairments to basic memory/perceptual deficits.

In addition to behavioural evidence for PS deficits, impairments in both excitatory and inhibitory dentate gyrus (DG) neurons are observed in schizophrenia. Schizophrenia is associated with decreased DG glutamate release^53^ which is partially due to decreased neurogenesis in this subfield^63^. The loss of interneurons in the DG is also implicated in PS deficits, as inhibition is critical for maintaining the sparseness of DG granule cell firing, a feature that is critical for pattern separation^64^. Reduced glutamatergic transmission from DG neurons to CA3 has also been proposed to enhance PC by triggering metaplasticity mechanisms that could lower the threshold for LTP at inputs onto CA3, including those from synapses made by entorhinal cortical inputs and recurrent CA3-CA3 synapses^52^. This would in turn favour the PC mediated by such recurrent connections^52^. Consistent with this, post-mortem analysis of schizophrenia tissue reveals increased GluN2B-containing NMDA receptors (GluN2B/GluN1) in CA3 tissue, but not in CA1^54^. Given the preferential involvement of such receptors in the induction of long-term potentiation^65^, this may point to an enhanced capacity for LTP at CA3-CA3 synapses. However, while these results are consistent with an enhanced PC, they do not provide direct support. The loss of PV interneurons, which is also seen in area CA3^48^ may contribute to enhanced pattern completion, by shifting the excitatory-inhibitory balance of CA3 networks towards excitation, and hence promoting aberrant Hebbian associations during memory formation and/or retrieval (Fig. 2; Supplementary Discussion 1). This would have the effect of reducing the threshold for jumps between attractors during memory retrieval (Fig. 1). Studies of both synaptic plasticity at CA3-CA3 synapses, dynamics of CA3 neuronal ensembles during retrieval, and behavioural expression of pattern completion in patients and animal models are needed to establish whether PC is enhanced in schizophrenia patients.

#### Pathway to shallow attractors

An imbalance of PC/PS tipped against PS would result in forming inappropriate associations between unrelated memories. The threshold for traversing neural states representing such memories is therefore lowered, giving rise to shallow attractors and disorganized cognitive maps.

### Aberrant salience

Inappropriate associations and disorganized cognitive maps in schizophrenia may arise due to aberrant salience: the process in which irrelevant stimuli are attributed significance^66^. Subjects at high risk of psychosis attribute greater relevance to irrelevant cues^67^. A prominent theory, Wagner’s model of stimulus processing, suggests that such increased attention to stimuli results from a decreased decay of stimuli from the primary active state (a state of maximal attention, typically reserved for highly salient and novel stimuli) to the secondary active state (a state of lower attention, typically occupied by more familiar stimuli)^68^. Such persistence in a highly active state promotes aberrant associations linking irrelevant stimuli^69^.

**Fig. 3:**
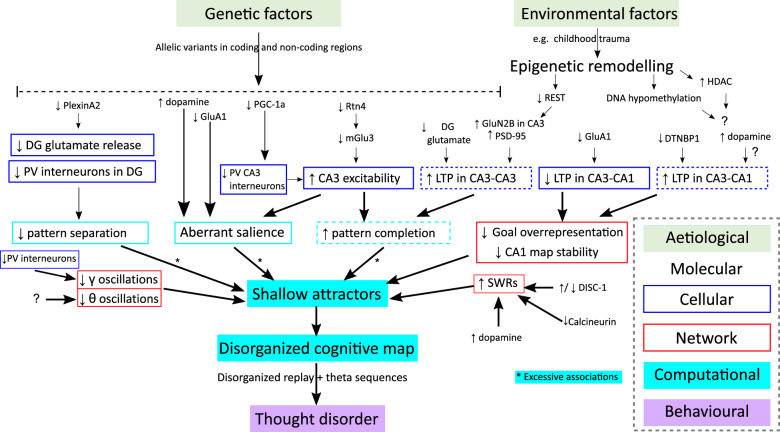
Convergence of distinct Hippocampal pathways on Thought disorder. Multiple pathways can give rise to thought disorder, either separately or in concert. Both genetic and epigenetic factors contribute to molecular deficits in key Hippocampal circuit elements. This in turn gives rise to deficits in the activity and plasticity of the cells affected. Such functional changes drive distinct network deficits which all converge on shallowing of Hippocampal attractors and hence disorganizing cognitive maps. Cognitive map disorganization is reflected in impaired sequential activity of Hippocampal place cells in offline and online states, which we propose drives thought disorder. Dashed lines indicate processes tentatively/indirectly implicated in Schizophrenia. (HDAC Histone Deacetylases, REST repressor element-1 silencing transcription factor, SWRs Sharp-wave ripples, PV Parvalbumin; asterisks (*) signify changes that cause excessive associations).

Aberrant salience has been reported in a mouse model in which the schizophrenia risk gene (*GriA1*) is selectively ablated^69^. This gene encodes the GluA1 subunit of AMPA receptors, which is implicated in Hippocampal plasticity, especially long-term potentiation^70^. GluA1 knockout animals show deficits in stimulus-specific short-term habituation and increased attention to neutral stimulus i.e., aberrantly ascribing importance to a non-significant event^55^. Crucially, these mice are more readily able to form associations between stimuli^71^ and long-term memories^71^ compared to wildtype controls. Reintroduction of GluA1 selectively into CA2/CA3 restored abnormal short-term habituation as well as the power of, and coherence between, Hippocampal and prefrontal theta oscillations^55^. This implicates CA3 function and plasticity in aberrant salience (Fig. 3; Supplementary Discussion 1). Indeed, recent evidence suggests that such Hippocampal dysfunction is a cause for, rather than the effect of, the hyperdopaminergic state in schizophrenia^72^. Another recent study found aberrant salience in mice exhibiting a distinct genetic alteration, knocking out PGC-1a KO mice, a gene implicated in schizophrenia and encoding a transcriptional coactivator^73^. These mice exhibited deficits in short-term habituation and decreased PV interneurons in areas CA3 and CA1. Thus, distinct genetic alterations seem to converge on a hyperactive CA3, which seems critical for driving both aberrant salience and the hyperdopaminergic state in schizophrenia. The circuit mechanisms by which these Hippocampal (especially CA3) deficits mediate aberrant salience, and its relationship (or lack thereof) to imbalanced PS and PC remains an active area of investigation.

#### Pathway to shallow attractors

Attributing salience to neutral or irrelevant stimuli promotes excessive associative plasticity and hence excessive associations. Similar to PS/PC imbalance, this would reduce the threshold for transitions between attractors for unrelated memories in the neural state space and hence result in disorganized cognitive maps.

### Aberrant goal attractors in the CA1

Salient locations associated with reward, cues, or safety, are represented by more place cells than non-salient locations in the CA1, suggesting a reorganisation of the map towards goals^74–76^. Such a phenomena was predicted by early continuous attractor models of Hippocampal function, and proposed to arise from enhanced synaptic plasticity at goal locations^14^. Intriguingly, in the Df(16)A^+/−^ mouse model of schizophrenia, the overnight stability and reward-related stabilisation of CA1 spatial maps was reduced and there was a marked lack of reorganisation of place cells towards goal locations^36^ (Figs. 2, 3; Supplementary Discussion 1). This may reflect a general phenomenon of shallow attractors in schizophrenia, whereby reduced CA3-CA1 plasticity results in impaired stabilization of assemblies representing goals and paths towards them (Supplementary Discussion 1). Indeed, systemic pharmacological blockade of NMDA-receptors abolishes goal overrepresentation in CA1 and impairs goal-directed behaviour during memory retrieval^75^. Alternatively, shallowing of goal attractors may arise from enhanced plasticity (Supplementary Discussion 1), as predicted by the aberrant salience hypothesis of schizophrenia. Inappropriately attaching importance to non-goal locations would result in a net under-representation of experiences of true salience and perhaps an inability to recall those when prompted. If everything is a goal, then nothing is a goal.

#### Pathway to shallow attractors

Reduced synaptic/network plasticity could reduce stabilization of assemblies representing salient states, and hence reduce the deepening of attractors for these states. On the other hand, an enhanced but non-specific (goal-independent) plasticity may also lead to a *relative* shallowing of attractors, as neural states representing non-salient and/or unrelated items are equally deepened. In both cases, the network would fail to converge on the appropriate goal-state during memory retrieval, instead aberrantly transitioning between irrelevant states in a disorganized manner. This framework puts the emphasis on attractor landscape and network dynamics, rather than the direction of the activity or plasticity impairment, in mediating thought disturbance in schizophrenia.

We note that, while goal-directed behaviour (including speech) is more readily measurable in patients and animal models, shallowing of the attractor landscape representing a given state-space should cause frequent jumps between attractors representing distinct relational sets regardless of the degree to which behaviour/thought is guided by an explicit goal. This would manifest as increased derailment of speech in the absence of obvious cues or a “topic” of conversation. It will be intriguing to investigate whether there are sub-stratifications amongst schizophrenic patients, with some patients showing tangential speech only when speech is goal-directed (e.g. when answering a specific question) but not derailment with more unconstrained speech. Such stratifications may correlate with distinct patterns of neurobiological impairments (e.g. distinct involvement of goal-related Hippocampal–prefrontal interactions).

### Hippocampal–cortical interactions

While our focus here is on Hippocampal pathologies, it is important to contextualise these deficits in light of well-established cortical pathologies associated with the disease. In particular, impairments in brain regions implicated in semantic processing, including frontal (e.g. inferior frontal gyrus) and temporal (e.g. superior temporal gyrus (STG)) regions, have been strongly implicated in formal thought disorder^77,78^. How are Hippocampal impairments related to those in cortical areas and to semantic processing? Hippocampal-cortical synchrony during theta/gamma oscillations and sharp wave ripples has been implicated in cognitive mapping functions in rodent and human studies^39,79^. Given the impairments in these network dynamics in schizophrenia^34–36,79^, it is plausible that dysregulated synchrony between Hippocampus and cortical language areas results in aberrant activity and plasticity across these regions, which could be part of the mechanism driving thought disorder. Indeed, a recent study links Hippocampal theta power to semantic processing and implicates theta synchrony between Hippocampus and STG in this process^80^. Furthermore, pre-retrieval Hippocampal theta power correlates with distances in semantic space^81^, suggesting analogies between the encoding of conceptual and physical distances in Hippocampal activity. These studies^80,81^ are consistent with the view that Hippocampal cognitive mapping could extend to mapping semantic structures through theta-mediated interactions with semantic-network areas in the temporal cortex. Disrupted mapping of such semantic distances would in turn provide a mechanism for the derailment and tangentiality seen in positive formal thought disorder. Detailed fMRI-based representational analysis across these regions in patients and controls will be critical to address this possibility.

In addition, communication between the Hippocampus and prefrontal cortex (PFC), such as that mediated by theta oscillations, is also impaired in Schizophrenia^79^. Such interactions may be particularly important for goal-directed thought or speech, given the critical role of Hippocampal prefrontal communication in goal-directed behaviour in humans and animal models^82^. Overall, while such studies provide key pointers, further studies are needed to synthesize such literature into a coherent computational and mechanistic account of how Hippocampal–cortical interactions relate to semantic and goal-related impairments in thought disorder.

### Prediction 3 - Molecular changes give rise to positive formal thought disorder as a function of their efficacy in shallowing Hippocampal cognitive maps

It is well established that schizophrenia in general^83^, and thought disorder in particular^84^, has a heritable component. In addition, epigenetic factors are strongly implicated in the aetiology of the disease^85^, providing an entry point into understanding environmental influences on Schizophrenia. Despite this, no single gene has been identified to be fully penetrant in generating Schizophrenia symptoms: Schizophrenia is a complex polygenetic disease. Efforts to understand the unifying principles underlying the involvement of distinct molecules and molecular pathways in the disease are therefore critical if its aetiology is to be understood. Genome-wide association studies (GWASs) and gene expression studies have particularly implicated genes related to synaptic function and plasticity^86–89^. However, lower level cellular processes (such as LTP or interneuron function) alone are insufficient to explain the development of thought disorder. These are both unspecific to the disease and often seemingly contradictory (e.g., both enhanced^51^ and impaired^49,50^ LTP reported at CA3-CA1 synapses in Schizophrenia; see Supplementary Discussion 1). Our framework instead emphasizes that a gene’s involvement in thought disorder is predicted by its contribution to the depth of Hippocampal attractors. Recent combinatorial genotyping-functional approaches can begin to speak to this. Data from Schizophrenia-related GWAS loci have been integrated with gene-gene network information under a Bayesian framework to better predict risk genes^90^. A large proportion of “high confidence” risk genes identified from this analysis were implicated in synaptic development and plasticity (including GluA1 and GluN2A subunits of AMPA and NMDA glutamatergic receptors respectively). Crucially, these genes were enriched in the Hippocampus and frontal cortices, consistent with defective cognitive mapping as a mechanism of Schizophrenia. Our framework predicts that:

***Functional grouping of genes based on their roles in deepening attractors involved in cognitive mapping processes (via promoting pattern separation, pattern completion, goal overrepresentation, transitive inference…etc) should allow a more accurate prediction of positive formal thought disorder prevalence than functional grouping based on lower level processes (e.g. LTP, interneuron function…etc)***

While this casts a wide net on a number of processes and pathways, it remains specific: unlike genes promoting deeper attractors, we predict that loss-of-function in genes promoting *shallowness* of attractors (e.g. genes whose function is to downregulate PVN numbers and activity) would be protective rather than promote thought disorder.

In order to functionally annotate risk variants highlighted by GWASs, a powerful experimental paradigm has been to investigate the physiological and behavioural effects of disrupting such genes in mouse models. This line of research has repeatedly implicated deficits in processes related to Hippocampal cognitive mapping (see Prediction 2 and Fig. 3). We have discussed one such gene (GriA1), encoding the AMPA receptor subunit GluA1, where gene knockout results in aberrant salience in a CA3-dependent manner under “Prediction 2” above. Other gene knockouts have also yielded insight into the contribution of distinct Hippocampal computations. For instance, GWAS data identified *Rtn4* as a risk gene associated with Schizophrenia. Knockout of its functional protein Nogo-A in mice led to hyperexcitability in CA3 circuits and downregulation of mGlu3 metabotropic glutamate receptors^91^. Such hyperactivity may potentially give rise to the enhancement in pattern completion hypothesized to be one mechanism for thought disorder in Schizophrenia (Fig. 2)^52^. While this possibility was not tested, this study did show a behavioural correlate: knockout mice relied more heavily on global (compared to local) reference frames when performing the Morris Water Maze task. Testing memory retrieval performance in the presence of only partial cues would provide a more explicit test of pattern completion in this mouse model, and potentially open avenues for relating gene variants to behavioural stratification of the disease. Moreover, mutations in the plexin family (Plxna2/Plxna4) of axon guidance molecule receptors are implicated in schizophrenic patients^92^. Knockout of PlexinA2 in mice results in impaired DG development, neurogenesis, and DG→CA3 synaptic connectivity^93^. This may in turn drive the pattern separation deficits seen in Schizophrenia patients (see Prediction 2). The same study showed that mice exhibited deficits in Hippocampus-dependent contextual (but not cued) fear conditioning. Nevertheless, a direct demonstration of a pattern separation deficit remains a future prospect.

In addition to the heritable component of Schizophrenia, environmental factors are critical in disease aetiology. Epigenetic alterations may provide a mechanistic explanation for the role of such environmental factors^94–96^. Life events such as childhood trauma are associated with disrupted transcriptional regulation such as hypomethylation of repetitive DNA sequences^97^ and increases in histone deacetylase expression^98^ in schizophrenic patients. These changes have been associated with the plasticity and development of Hippocampal circuits^99,100^. Can we link such epigenetic changes to Hippocampal cognitive mapping? One promising line of evidence involves the ubiquitous gene repressor, repressor element-1 silencing transcription factor (REST). REST is involved in the age-related maturation of the NMDA receptor from GluN2B- to GluN2A-containing NMDA receptors through epigenetic remodelling^101^. Reduced REST activity in Schizophrenia patients coincides with increased GluN2B-containing NMDA receptors in CA3, along with increased postsynaptic density protein-95 (PSD-95) and augmented dendritic spines on the pyramidal neuron apical dendrites^101^. This demonstrates a potential epigenetic basis for markers of increased LTP in CA3. If validated with functional plasticity and behavioural studies, this would suggest a link between epigenetic changes and increased pattern completion, disorganized cognitive maps, and consequently, thought disorder. While such a mechanistic link is currently largely speculative, what is clear is that life events, particularly childhood trauma, are linked to Hippocampal changes in Schizophrenia^102^. By combining this line of work with findings implicating synaptic plasticity genes in Schizophrenia, we can investigate the extent to which environment-induced epigenetic changes may converge on deficits in synaptic and cellular plasticity underlying the formation of Hippocampal cognitive maps.

Overall, evidence is beginning to shed light on distinct molecular changes that converge on the shallowing of Hippocampal attractors. A more definitive picture will emerge from (i) stratification of GWAS/transcriptomic results based on the prevalence (and type) of thought disorder and (ii) annotation of molecular function using a combination of neural ensemble recordings and explicit cognitive tests of cognitive mapping processes (such as pattern separation, pattern completion and transitive inference) in patients and animal models.

### Implications for treatment

The cognitive mapping framework we explore here provides grounding for a combinatorial approach to treating Schizophrenia. Across a multitude of psychiatric disorders, it is becoming clear that a combination of pharmacological interventions and behavioural/psychological rehabilitation (e.g., via cognitive behavioural therapy) might be a highly effective way of alleviating symptoms or even preventing disease onset in the first place^103^. In the context of Schizophrenia, we suggest that understanding thought disorder as a shallowing of cognitive maps allows a more targeted approach on both the drug-based and behavioural fronts (Supplementary Discussion 2). Moreover, understanding that multiple heterogenous aetiological streams could converge on producing shallow, disorganized cognitive maps (Fig. 3) could allow more personalized interventions (Supplementary Discussion 2).

### Conclusions and future directions

Our framework emphasizes shallow attractors underlying Hippocampal cognitive maps as a core mechanism for positive formal thought disorder in Schizophrenia. This unifies several theories of the disorder and promises to explain the heterogeneous aetiology of the disease. We propose that multiple distinct pathologies converge on shallowing of Hippocampal attractors. We have outlined some key predictions from this framework and summarised evidence that pertains to each (see Supplementary Discussion 3 for additional detailed experimental predictions arising from our framework).

Our focus here has been on Hippocampal pathology and thought disorder. However, Schizophrenia pathology is not restricted to the Hippocampus, nor to thought disorder. Multiple related regions are implicated, most notably the medial, orbital, and dorsolateral PFC^10,11^ as well as the STG^77,78^. The role of Hippocampus in relational mapping of multiple analogous spaces^8,104–106^, and its strong interconnectivity with cortical regions means that Hippocampal pathology may be closely related to such cortical pathologies. It will therefore be important to investigate the following questions:

### (1) To what extent is shallowing of attractors in a given brain area causally linked to specific impaired computations and hence symptoms?

For example, does shallowing of attractors in dorsolateral PFC specifically drive working memory impairments^107^ while shallowing of Hippocampal attractors causes disorganized cognitive mapping and positive formal thought disorder?^32^ Moreover, the medial and orbital PFC have been ascribed their own cognitive mapping functions, providing abstract maps of “task-space”^108,109^ and exhibiting temporally organized “replay” analogous to that seen in the Hippocampus^37^. Analogous shallowing of attractors underlying medial and/or orbital prefrontal maps may contribute to disorganization of such abstract maps, perhaps giving rise to distinct types of symptoms (e.g. delusional beliefs). These anatomical dissociations could provide some explanation for the heterogeneity and dissociability of the distinct symptoms expressed in Schizophrenia.

### (2) How do impaired interactions between Hippocampus and a given cortical area contribute to thought disorder and the other symptoms of the disease?

As well as the potential role of Hippocampal-STG communication in mapping semantic spaces (discussed above under “Prediction 2: Hippocampal-cortical interactions”), theta-mediated interactions between the Hippocampus and the mPFC have been implicated in cognitive mapping^82^ and reduced in Schizophrenia^79^. Prefrontal task maps are proposed to interact in rich and as yet poorly understood ways with Hippocampal relational maps to mediate flexible, model-based behaviour^82^. Investigating the functional implications of cross-region interactions in healthy subjects and animal models will allow generating precise hypotheses about the relationship between cross-circuit interactions and symptoms, including thought disorder.

### (3) Could shallowing of attractors and the resulting dysregulated activity in one area (e.g. Hippocampus) drive shallowing in another connected area implicated in the disease (e.g. mPFC) through aberrant activity-dependent cross-regional plasticity?

This would provide one mechanism for the common co-occurrence of distinct symptoms (such as delusions and thought disorder) in the disease. Moreover, the reciprocal connectivity between Hippocampus and cortical regions may drive a positive feedback loop that results in progressive shallowing of attractors across connected regions and hence drive the more severe deficits in mnemonic processing associated with chronic disease^62^.

Investigating these questions at the cellular and circuit levels promises to provide biological grounding to computational models that aim to unify positive symptoms within a Predictive (Bayesian) framework^110,111^. Overall, while here we focus on Hippocampal cognitive mapping deficits, we anticipate that insights gleaned from this synthesis will yield broad and generalizable insights into the wider symptomatology and aetiology of the disease.

## Supplementary information

Supplementary Information
