# Supplementary material for: The shallow cognitive map hypothesis: A hippocampal framework for thought disorder in schizophrenia
Source: Schizophrenia (Heidelb). 2022 Apr 7;8(1):34. doi: 10.1038/s41537-022-00247-7 (PMC9261089; doi:10.1038/s41537-022-00247-7)
Supplement: Supplementary file 1 — Supplementary Information [file 41537_2022_247_MOESM1_ESM.pdf]

Supplementary Information

Supplementary Discussion 1 - Shallow Attractors unify chemical hypotheses of Schizophrenia

Explaining positive formal thought disorder in Schizophrenia as the result of shallow, disorganized Hippocampal cognitive mapping provides a framework for relating multiple, seemingly competing transmitter-based theories of Schizophrenia: the dopamine, GABA and glutamate hypotheses.

The Dopamine hypothesis

It is widely accepted that dopamine dysfunction plays a role in Schizophrenia[112]. A hyperdopaminergic state is closely associated with psychotic symptoms. While much of this has been attributed to the dopaminergic innervation of the basal ganglia via the nigrostriatal pathway, bidirectional interactions between dopaminergic cells in the ventral tegmental area (VTA) and the Hippocampus have also been implicated in the disease[71]. Excessive activity in both the Hippocampus and VTA may be part of a positive feedback loop where both structures mutually enhance, and dysregulate, each other's activity. Under **Prediction 2**, we discussed how Hippocampal dysfunction can drive the hyperdopaminergic state. Below we discuss how the hyperdopaminergic state could contribute to Hippocampal dysfunction.

Within this Hippocampus, dopamine is a key regulator of synaptic and network plasticity, particularly in relation to novelty or reward prediction errors[113]. Dopamine acting on D1-receptors enhances LTP and can alter Hippocampal spike timing dependent plasticity (STDP) rules, for example by retroactively converting spike-timing dependent LTD to LTP[114]. Increased tonic firing of dopaminergic afferents in Schizophrenia may underlie aberrant salience[115], potentially by reducing the threshold for LTP induction and resulting in excessive, inappropriate associations. At the network level, dopamine has been shown to facilitate the stabilization of spatial maps[116] and goal-representations[117] in the Hippocampus, both of which are impaired in a mouse model of Schizophrenia[36]. Dysregulated and excessive dopamine release may impair the selective stabilization of goal representations, giving rise to shallow attractors. A recent study suggests that

dopamine release via the Locus coeruleus may be critical for this effect[118]. Moreover, another recent study identifies dopaminergic locus coeruleus inputs to the dorsal CA1 as being critical for linking memories[119]. Assessing the effect of dopamine receptor blockade, or optogenetic manipulation of dopaminergic afferents, on Hippocampal attractor dynamics in Schizophrenia models would shed further light on the relationship between dopaminergic and hippocampal dysfunction in thought disorder.

The GABA hypothesis

In addition to dysregulated dopamine, there is robust evidence to support an association between loss of Hippocampal interneurons and Schizophrenia. Hippocampal interneuron deficits have been reported in Schizophrenia patients[48,120,121], and ablation of NMDAR receptors in corticolimbic interneurons induces Schizophrenia-like symptoms in mice[122]. Moreover, as discussed above, the loss of parvalbumin interneurons in the DG has been implicated in pattern separation effects in Schizophrenia. More generally, an impaired Excitatory inhibitory balance in the Hippocampus may underlie excessive associations in Schizophrenia[123,124]. Parvalbumin positive interneurons (PVNs), which receive converging input from principal neurons and in turn target the somatic and perisomatic compartments of such neurons, are critical for temporally organizing activity within a given neural assembly, and demarcating activity across assemblies (**Figure 2**)[16,125]. Disrupting such a process would results in increased overlap between neural ensembles encoding distinct contexts and may underlie disorganized maps in Schizophrenia. Consistent with this, Belforte et al[122] showed that knockout of NMDARs in cortico-limbic PVNs before 8 weeks, results in a schizophrenic phenotype (e.g. novelty induced hyperlocomotion) and the attenuation of gamma oscillations (However, see Bygrave et al 2016[126] for confounding factors that suggest NMDAR hypofunction involves a multitude of neuron types). Moreover, Gamma oscillations, known to be deficient in Schizophrenia patients and models, are generated by a mechanism that involves feedback inhibition by fast-spiking PV interneurons (PVN)[127]. The importance of early disruption of these circuits and the role of PVN has also been highlighted in a recent study where schizophrenic behaviours (novel object recognition, sensory-motor gating in Paired pulse inhibition) in *PV*-Cre; *ErbB4*$^{fl/fl}$ mice could be reversed by

chemogenetic activation of PVN in the frontal association cortex[128]. A similar mechanism may occur in the Hippocampus where impairments in PVN have been implicated in schizophrenic patients and mouse models. Indeed, post-mortem staining of schizophrenic brains have shown a reduction in Hippocampal PVN density compared to controls[48] (but see Woo et al [129]) and more recent transcriptomics studies point to functional differences in neocortical PVNs amongst patients and controls[130]. These findings are consistent with loss/dysfunction of PVN mediating disorganized neuronal ensemble activity in Schizophrenia. Direct evidence would entail testing whether Hippocampal neural ensembles encoding distinct memories are disorganized in Schizophrenia in a manner that is dependent on PVN dysfunction.

The Glutamate hypothesis

That NMDA receptor antagonists, such as phencyclidine (PCP), induce or exacerbate psychotic symptoms in humans has led to the glutamate hypothesis of Schizophrenia[131,132]. This has informed mechanistic animal studies, where PCP induces psychosis-like symptoms and synaptic changes in the hippocampus and amygdala of rats[50]. In particular, NMDA receptor-dependent LTP is impaired in the CA1 of Schizophrenia animal models[49,50]. Deficits in LTP induction may underlie the inability to form stable attractors at goal locations in Schizophrenia. Consistent with this, pharmacological blockade of NMDA receptors prevents overrepresentation of goals in area CA1 (**Figure 2**)[75]. However, this manipulation was systemic and would have affected NMDA-dependent processes throughout the brain, including those unrelated to LTP. A direct link between LTP deficits and goal overrepresentation in the Hippocampus would require, for example, depotentiating synapses potentiated during learning and observing a loss of goal overrepresentation[133] . The relationship between plasticity and Schizophrenia may be more complex, with enhancements in LTP also reported in Schizophrenia models. For example, the Schizophrenia associated DTNBP1 gene encodes Dysbindin protein that plays a role in the neuronal trafficking of proteins including receptors. In dysbindin-deficient mice, enhanced AMPAR responses and LTP were observed in CA3-CA1 Hippocampal synapses[51]. Nevertheless, as discussed above, an enhanced potentiation, if it is not specific to neuronal inputs representing rewarded or salient states, may give rise to aberrant or dysregulated associations, which

would result in the same net effect of shallowing attractors and disorganizing Hippocampal maps (**Figure 3**).

Overall, these findings suggest that dysregulated dopamine, compromised interneuron function and aberrant plasticity at glutamatergic synapses could all contribute to the shallowing of Hippocampal attractors underlying cognitive map function in the Hippocampus. This framing is useful in two ways. Firstly, it provides a unified framework for understanding the role of these distinct transmitter systems in Schizophrenia. Secondly it provides a rationale for understanding the heterogeneity that famously characterizes the aetiology of Schizophrenia. Multiple distinct pathways could achieve a similar end result: a shallow, disorganized cognitive map.

Supplementary Discussion 2 - Avenues for treatment

Our framework provides rationale for the following:

1-Prognosis: In addition to Schizophrenia patients, relatives of schizophrenics also score higher on thought disorder severity than non-psychiatric controls[84]. Identifying mutations and epigenetic changes in Hippocampal plasticity-related genes that predispose individuals to disease not only allows identifying at-risk individuals and families, but also may allow more precise prediction of their probable disease progression and predisposition to thought disorder.

Example: Using model-based approaches to classify individuals as at risk of developing thought disorder based on the proximity of their genetic profiles to those of Schizophrenia datasets associated with this symptom, guided by functional data linking such genes to shallowing of Hippocampal cognitive maps.

2-Prevention: Point 1 above combined with understanding how epigenetic changes associated with early life events, such as traumatic life experiences, interact with genetic factors to elicit Hippocampal dysfunction can form the basis for precise, appropriately timed and personalized behavioural and drug-based interventions that prevent disease onset.

Example: molecular and environmental factors affecting early Hippocampal development (e.g. Plexin mutations, exercise, trauma) could be targeted in at risk individuals.

3-Treatment: Results showing mechanisms of symptom reversal in animal models provide hope for more precisely targeted post-hoc treatments when preventative measures fail.

Example: exercise[134] and environmental enrichment [128,135] can reverse Schizophrenia symptoms in animal models by enhancing interneuron neurogenesis or function in the dentate gyrus respectively. This provides a mechanistic understanding of exercise-induced improvements in Hippocampal function in Schizophrenia patients[136]. Our framework explains such an effect as a result of rescuing imbalance of PS/PC by enhancing the former, thereby promoting the formation of organized, deep cognitive maps. Combining such

behavioural therapies with pharmacological treatments that target PV interneurons could provide a *robust* therapeutic strategy, especially for non-responders to dopaminergic treatments.

Supplementary Discussion 3 - Additional Detailed Experimental Predictions

1-Impaired replay in Schizophrenia is the result of "jumps" between Hippocampal sequences representing distinct memories: e.g., in Schizophrenia mouse models experiencing two or more linear tracks, awake replay (in CA3 and CA1) in one track should exhibit a greater intermixing of non-random fragments of sequences from multiple tracks (**Figure 1b**).

2-Jumps between sequences for distinct memories may correlate with enhanced performance on tasks that require associating distinct memories, but should also impair tasks that necessitate suppressing memory interference. This pattern should be seen in human patients at early or subclinical stages of the disease (e.g., at risk relatives of patients).

3-Is thought disorder associated with dysregulated encoding or retrieval of relational maps?
An impairment in encoding of relational structures (e.g. due to excessive associations driven by aberrant salience) would involve anterograde deficits restricted to relational structures learned after disease onset. Conversely, retrograde deficits would indicate some level of retrieval impairment too. However, this is complicated by the difficulty in determining disease onset and the mounting evidence for premorbid cognitive deficits (e.g. [137]). It is also the case that memories are subject to modification both offline and when explicitly retrieved (memory reconsolidation[138]), which would mean that even an anterograde deficit could affect relational structures learned prior to disease onset. This could be resolved by more controlled experiments in which acute models of thought disorder are investigated (perhaps those involving ketamine induced psychosis, which is known to include thought disorder[139]). This would allow precise determination of impairment onset and better control of memory processing.

4-Molecular changes that impair pattern separation, enhance pattern completion or induce aberrant salience should have a similar end result of shallowing Hippocampal attractors in the CA3, causing jumps during awake replay (see point 1) and predisposing subjects to thought disorder (**Figure 3**). Functional grouping of genes based on their roles in deepening hippocampal attractors should allow a more accurate prediction of patient symptoms and prognosis than functional grouping based on lower level processes (e.g. LTP, interneuron function...etc). This may emphasize genes with promotors/regulatory elements that are preferentially transcriptionally activated in the Hippocampus.

5-Molecular changes that give rise to either impaired LTP or enhanced yet dysregulated LTP at synapses onto CA1 (including CA3-CA1 and nucleus reuniens-CA1 synapses) should effectively result in a net shallowing of attractors towards goals relative to non-goal locations - giving rise to impaired goal overrepresentation and goal-directed navigation. Where plasticity is enhanced, our framework predicts that this enhancement will exhibit little or no specificity for salient compared to non-salient stimuli. This may involve enhanced heterosynaptic plasticity, where synapses other than those exhibiting plasticity inducing patterns of activity are potentiated/depressed. Alternatively, this could involve a reduced threshold for inducing homosynaptic plasticity, such that weak patterns of activity that typically don't induce plasticity at a given synaptic input in controls are effective in driving plasticity in schizophrenic models. An enhanced LTP that maintains input specificity (e.g. if induction threshold is maintained but LTP expression is enhanced) should have the opposite effect, causing deeper attractors to form and hence being protective against thought disorder.

6-Cognitive-mapping-related changes outlined in points 1-5 should be enriched in Schizophrenia patients with a high conceptual disorganization score on the PANSS compared to those with lower scores.

Supplementary References

112. van Rossum, J. M. The significance of dopamine-receptor blockade for the mechanism of action of neuroleptic drugs. *Arch. Int. Pharmacodyn. Ther.* **160**, 492–494 (1966).

113. Sosa, M. & Giocomo, L. M. Navigating for reward. *Nat. Rev. Neurosci.* **22**, 472–487 (2021).

114. Brzosko, Z., Schultz, W. & Paulsen, O. Retroactive modulation of spike timing-dependent plasticity by dopamine. *Elife* **4**, (2015).

115. Grace, A. A. Dysregulation of the dopamine system in the pathophysiology of schizophrenia and depression. *Nat. Rev. Neurosci.* **17**, 524–532 (2016).

116. McNamara, C. G., Tejero-Cantero, Á., Trouche, S., Campo-Urriza, N. & Dupret, D. Dopaminergic neurons promote hippocampal reactivation and spatial memory persistence. *Nat. Neurosci.* **17**, 1658–1660 (2014).

117. Retailleau, A. & Morris, G. Spatial Rule Learning and Corresponding CA1 Place Cell Reorientation Depend on Local Dopamine Release. *Curr. Biol.* **28**, 836-846.e4 (2018).

118. Kaufman, A. M., Geiller, T. & Losonczy, A. A Role for the Locus Coeruleus in Hippocampal CA1 Place Cell Reorganization during Spatial Reward Learning. *Neuron* **105**, 1018-1026.e4 (2020).

119. Chowdhury, A. *et al.* A Locus Coeruleus- dorsal CA1 dopaminergic circuit modulates memory linking. Preprint at https://www.biorxiv.org/content/10.1101/2021.10.27.466138v1.full (2021).

120. Benes, F. M., Kwok, E. W., Vincent, S. L. & Todtenkopf, M. S. A reduction of nonpyramidal cells in sector CA2 of schizophrenics and manic depressives. *Biol. Psychiatry* **44**, 88–97 (1998).

121. Konradi, C. *et al.* Hippocampal interneurons are abnormal in schizophrenia. *Schizophr. Res.* **131**, 165–173 (2011).

122. Belforte, J. E. *et al.* Postnatal NMDA receptor ablation in corticolimbic interneurons confers schizophrenia-like phenotypes. *Nat. Neurosci.* **13**, 76–83 (2010).

123. Lewis, D. A., Hashimoto, T. & Volk, D. W. Cortical inhibitory neurons and schizophrenia. *Nat. Rev. Neurosci.* **6**, 312–324 (2005).

124. Vogels, T. P. & Abbott, L. F. Gating deficits in model networks: a path to schizophrenia?

Pharmacopsychiatry **40 Suppl 1**, S73-7 (2007).

125. Buzsáki, G. Neural syntax: cell assemblies, synapsembles, and readers. *Neuron* **68**, 362–385 (2010).

126. Bygrave, A. M. *et al.* Knockout of NMDA-receptors from parvalbumin interneurons sensitizes to schizophrenia-related deficits induced by MK-801. *Transl. Psychiatry* **6**, e778 (2016).

127. Butler, J. L. & Paulsen, O. Hippocampal network oscillations - recent insights from in vitro experiments. *Curr. Opin. Neurobiol.* **31**, 40–44 (2015).

128. Huang, Y. *et al.* Environmental enrichment or selective activation of parvalbumin-expressing interneurons ameliorates synaptic and behavioral deficits in animal models with schizophrenia-like behaviors during adolescence. *Mol. Psychiatry* (2021). doi:10.1038/s41380-020-01005-w

129. Woo, T. U., Miller, J. L. & Lewis, D. A. Schizophrenia and the parvalbumin-containing class of cortical local circuit neurons. *Am. J. Psychiatry* **154**, 1013–1015 (1997).

130. Enwright Iii, J. F. *et al.* Transcriptome alterations of prefrontal cortical parvalbumin neurons in schizophrenia. *Mol. Psychiatry* **23**, 1606–1613 (2018).

131. Bygrave, A. M., Kilonzo, K., Kullmann, D. M., Bannerman, D. M. & Kätzel, D. Can N-Methyl-D-Aspartate Receptor Hypofunction in Schizophrenia Be Localized to an Individual Cell Type? *Front. psychiatry* **10**, 835 (2019).

132. Kim, J. S., Kornhuber, H. H., Schmid-Burgk, W. & Holzmüller, B. Low cerebrospinal fluid glutamate in schizophrenic patients and a new hypothesis on schizophrenia. *Neurosci. Lett.* **20**, 379–382 (1980).

133. Hayashi-Takagi, A. *et al.* Labelling and optical erasure of synaptic memory traces in the motor cortex. *Nature* **525**, 333–338 (2015).

134. Yi, Y., Song, Y. & Lu, Y. Parvalbumin Interneuron Activation-Dependent Adult Hippocampal Neurogenesis Is Required for Treadmill Running to Reverse Schizophrenia-Like Phenotypes. *Front. cell Dev. Biol.* **8**, 24 (2020).

135. Guo, N. *et al.* A sensitive period for GABAergic interneurons in the dentate gyrus in modulating sensorimotor gating. *J. Neurosci.* **33**, 6691–6704 (2013).

136. Pajonk, F.-G. *et al.* Hippocampal plasticity in response to exercise in schizophrenia. *Arch. Gen.*

Psychiatry **67**, 133–143 (2010).

137. Allen, D. N., Kelley, M. E., Miyatake, R. K., Gurklis Jr., J. A. & van Kammen, D. P. Confirmation of a Two-Factor Model of Premorbid Adjustment in Males with Schizophrenia. *Schizophr. Bull.* **27**, 39–46 (2001).

138. Nader, K. Reconsolidation and the Dynamic Nature of Memory. *Cold Spring Harb. Perspect. Biol.* **7**, a021782–a021782 (2015).

139. Adler, C. M., Goldberg, T. E., Malhotra, A. K., Pickar, D. & Breier, A. Effects of ketamine on thought disorder, working memory, and semantic memory in healthy volunteers. *Biol. Psychiatry* **43**, 811–816 (1998).
